# The pathway from domestic remittances to food security in India: is agriculture a mediator?

**DOI:** 10.1186/s40066-025-00574-9

**Published:** 2026-03-22

**Authors:** Reshma P. Roshania, Regine Haardörfer

**Affiliations:** 1 National Council of Applied Economic Research, New Delhi, India; 2 Behavioral, Social, and Health Education Sciences, Rollins School of Public Health, Emory University, Atlanta, USA

**Keywords:** Agriculture, Remittances, Internal migration, Food security, Structural equation modeling, India

## Abstract

Remittances sent from migrants to agricultural origin households can improve food security through market purchases as result of increased income, improved production and consumption of home-produced food enabled by agricultural investments, or a combination of both. These mechanisms have thus far only been hypothesized. Using two waves of the India Human Development Survey (IHDS), we conducted a serial mediation analysis to test how agricultural investments, production, and consumption of home-grown foods mediate the pathway from domestic remittances to food security. Our results demonstrate that there are no differences between remittance and non-remittance receiving households in agricultural expenses or farm income, controlling for land size and other covariates. The total effects of remittances lower food insecurity; however, this is entirely explained by the market pathway, represented by the direct effect of remittances on food insecurity. Our findings challenge the policy narrative that migration lessens production. Policies that emphasize crop diversification to improve food security among rural smallholders must be migration sensitive. For households engaging in migration, policy imperatives include strengthening rural markets, especially for women-headed households, and making healthy foods affordable.

**Supplementary Information:**

The online version contains supplementary material available at 10.1186/s40066-025-00574-9.

## Introduction

International remittances, or money and goods sent by migrants to individuals residing in origin nations, are the largest source of external financing to low- and middle-income countries (LMICs) [1]. India has been the top global receiver of international remittances for over the past 15 years, and is the only country in the world to cross the 100 billion United States dollar (USD) mark of inflows [1]. Given the significance of international remittances to development, there is a strong body of research and robust data systems on the value and impact of the international remittance economy.

In India, however, most of the migration movement is internal to the country. Compared to the 17.9 million Indians who are abroad as of 2020 [2], official sources estimate that there are over 450 million internal migrants within India [3]; in addition to being exceedingly outdated, this figure does not include the over 100 million who engage in short-term and circular migration annually [4]. Current domestic remittance estimates are also unavailable; in 2007, they were calculated to be around 10 billion USD [5]. Nevertheless, it can be argued that in the Indian context, domestic remittances have a greater importance to household welfare compared to international remittances for two primary reasons: (1) 10% of households in India receive domestic remittances, compared to 1% of households that receive international remittances, and (2) 80% of domestic remittances are sent directly to rural households, compared to only 20% of international remittances. The majority of international remittances are directed towards migrants’ own financial investments, such as real estate, and thus do not constitute a critical component of origin families’ livelihood strategies [6]. Indeed, among households that receive money from internal migrants, remittances finance on average 30% of consumption costs [5], contribute to higher consumption in health, education and consumer durables [7], and can reduce household poverty [8].

India has experienced reductions in poverty, yet food insecurity remains high; a healthy diet, which meets nutritional requirements and food group diversity targets, is out of reach for 74% of the population [9]. Ensuring rural food security must recognize the role of migration and remittances as an essential household strategy, rather than solely focusing on agricultural production as the solution [10]. Indeed, over half of rural households in India are classified as agricultural^1^ [11]; relatively poor states with higher than average dependency on agriculture, such as Uttar Pradesh, Madhya Pradesh, Chhattisgarh, and Rajasthan, where, for example, 74% of households are agricultural, also experience high outmigration, demonstrating the criticality of multilocational livelihood strategies for rural households. In addition to the joint family structure, relatively cheap transport and communication systems, informality of work opportunities, and urban environments that are hostile to migrants, smallholdings are a critical factor as to why households do not move as an entire unit [12]. Rather, circular patterns of movement undertaken by only one or a few members of the household who remit money is a salient feature of internal migration in India.

Globally, there is evidence to suggest that remittances can increase food consumption, and improve food security and dietary diversity [13]. Similarly, in India, national level studies have found that an increase in remittances reduces food expenditure share—a measure of food insecurity, and raises overall food consumption, including that of nutrient-rich animal source foods, such as dairy, eggs, fish, and meat [14, 15]. Studies that have specifically distinguished between domestic and international remittances have been conducted in select states and have found a positive effect of international remittances on reduction of food share, while the difference in food share between domestic remittance receivers and non-remittance receivers was little to none [7, 16].

### Hypothesized pathways and theoretical underpinnings

The extant literature has not tested the mechanisms of the linkages between remittances and food security. The role of agriculture in the remittances to food security pathway has thus far only been hypothesized. Among households engaging in agriculture, can remittances result in improved food security via agricultural investments and changes in land use?

Remittance expenditures can influence change in land use and food security through several multi-directional modes. The loss of labor due to migration of household members can result in lower agricultural production and income [17], thereby lowering food security. This pathway aligns with early neo-classical migration theory, explained by individual decisions that result in a unidirectional societal transition from rural agriculture to urban industry [18]. Conversely, the new economics of labor migration (NELM) theory considers the household as the decision-making unit, and thus migration of one or more members is a livelihood strategy that diversifies risk and income for the family [19]. NELM makes explicit the linkages migrants maintain in source areas and the reciprocal effects of migration on development in the origin; remittances are a key aspect of this bidirectional flow. Applying NELM to the migration–agriculture–food security pathway suggests that agricultural investments enabled by remittances can improve production. Income from remittances can result in a shift from lower value subsistence crops to higher value commercial crops, can be used to hire labor or labor-saving technology, such as machinery and irrigation infrastructure, and can purchase or rent additional land [20]. Peanut farming households in Haiti that received remittances were more likely to use tarps for drying peanuts post-harvest, an important intervention for reducing aflatoxin prevalence, thereby improving yield, compared to non-remittance receiving households [21]. Changes in agricultural practices can, in turn, influence food security through a combination of production and income pathways, as has been laid out in existing conceptual agriculture–nutrition frameworks [22, 23]. This study aims to connect these pathways to explore how the relationship between domestic remittances and food security is mediated by agricultural investments, agricultural income, and consumption of home-grown food. This analysis can enable an understanding of the relative influence of agricultural versus market pathways to food security among rural households that employ multilocational livelihood strategies. The results have important policy implications for food security interventions in the agriculture, livelihood, and market sectors.

## Methods

### Data source and variables

We used India Human Development Survey (IHDS) [24] data from waves 1 (2004–05) and 2 (2011–12) for these analyses. The IHDS is a nationally representative, multi-topic, panel data set. Of the 40,018 households that were included in both waves, we removed households that received remittances from members who migrated internationally in either wave (*n* = 726).

Remittance status was coded as a dichotomous variable, indicating whether the household received any domestic remittances from a non-resident migrant member of the household in the previous 12 months.

Food consumption data for the previous 30 days were collected as a part of IHDS’ household consumption and expenditure module; consumption, including food produced at home, was summed across several food categories, including rice, wheat, pulses, vegetables, milk, meat, processed foods, food consumed outside of the home, etc. Monthly household food consumption share was used to create the dichotomous food insecurity variable. A food share of greater than 65% of total monthly consumption is considered high food insecurity [25].

IHDS captures a variety of farm and livestock expenses over the previous 12 months, such as hired labor, seeds, fertilizers, equipment, animal purchases, etc. The natural log of the sum of these expenses was used for analyses. The same approach was used for farm and livestock gross income from crop harvest and animal product sales over the previous 12 months.

Consumption of home-grown foods was coded as a dichotomous variable, indicating whether in the previous month, the household consumed any grains, pulses, vegetables, fruits, dairy, eggs, poultry, meat, oils or sweeteners that was grown or produced by the household.^2^ A value of zero implies that all foods were purchased through the market.

Several variables that are associated with both migration and food security and that have been used in the existing literature [14, 15] were included in the analyses as control variables; these are social group (a composite of caste^3^ and religion), state of origin household residence, urban or rural location of origin household, per capita income (log), gender of the head of the household, highest education level achieved in the household (none; 1–5 years; 6–10 years; and 11–14 years; bachelor degree or higher), dependency ratio (the number of adults 60 years and older and children 20 years and younger to the number of 21–59 years), ration card usage (dichotomous), farm size (none; smallholder—less than 2 hectares; 2 hectares or greater), and animal ownership (dichotomous).

### Statistical analysis

We first present descriptive statistics from the 2011–12 wave of sociodemographic characteristics, remittance status, and food production and consumption variables.

To explore the association between remittance status and food insecurity, we ran a lagged dependent variable logistic regression, with the following model specification:

$$\mathrm{ln}{\left(\frac{P}{1-P}\right)}_{2012}= {\beta }_{0}+ {\beta }_{1}Remittanc{e}_{2012}+{\beta }_{k}{{X}_{k}}_{2012}+\alpha FoodShar{e}_{2005}+{\varepsilon }_{2012}$$

where $\mathrm{ln}{\left(\frac{P}{1-P}\right)}_{2012}$ is the natural log of the odds ratio of food insecurity in the second wave, $Remittanc{e}_{2012}$ is the remittance status in the second wave, $FoodShar{e}_{2005}$ is the food consumption share of total household monthly consumption in the first wave, and ${{X}_{k}}_{2012}$ is the vector of control variables from the second wave.

Since lower food consumption share, and hence lower food insecurity, could be observed simply if a household reduces food quantity and/or quality while keeping total monthly expenditure constant, we additionally regressed per capita log monthly food consumption on remittance status to examine if overall, monthly food consumption is higher among households that began receiving remittances, controlling for the household’s prior inflation adjusted monthly food consumption (i.e., the lagged dependent variable), income, and other covariates mentioned. Exponentiated coefficients are presented for ease of interpretation. Descriptive statistics and lagged variable regressions were conducted in Stata.

### Serial mediation model

To examine the mechanisms of remittances and food insecurity, we first calculated polychoric, polyserial, and Pearson correlation coefficients among the study variables to understand the correlation between pairs of categorical–categorical, categorical–continuous, and continuous–continuous variables, respectively. We then conducted a serial mediation analysis [26] utilizing a generalized structural equation model. This method allows us to explore the hypothesized causal mechanisms between remittances (the exogenous variable) and food security, by measuring the extent to which agricultural investment, productivity, and consumption of produced foods explain this relationship; this is achieved through a series of equations regressing each endogenous variable in the pathway to obtain direct, indirect and total effects. In other words, the method allows assessment of the direct effect of the exogenous variable on the intermediate variables as well as the outcome variable, and the indirect effect of the exogenous variable on the outcome variable *through* intermediate variables. The generalized form of the model is suitable for non-linear variables.

Since it would be challenging to attribute change in food security in 2011–12 to remittances received in 2004–05, we only used data from the 2011–12 wave; nonetheless, we exploited data from 2004–05 by dropping households who received remittances in wave 1 (*n* = 1,723) so that we could compare those who never received remittances to those who began receiving remittances before 2011–12 but after 2004–05. In addition, temporality assumptions are met due to the reference periods of the model variables. We further restricted the analysis to agricultural households, defined as those households that cultivate land and/or own livestock (*n* = 21,142).

**Fig. 1:**
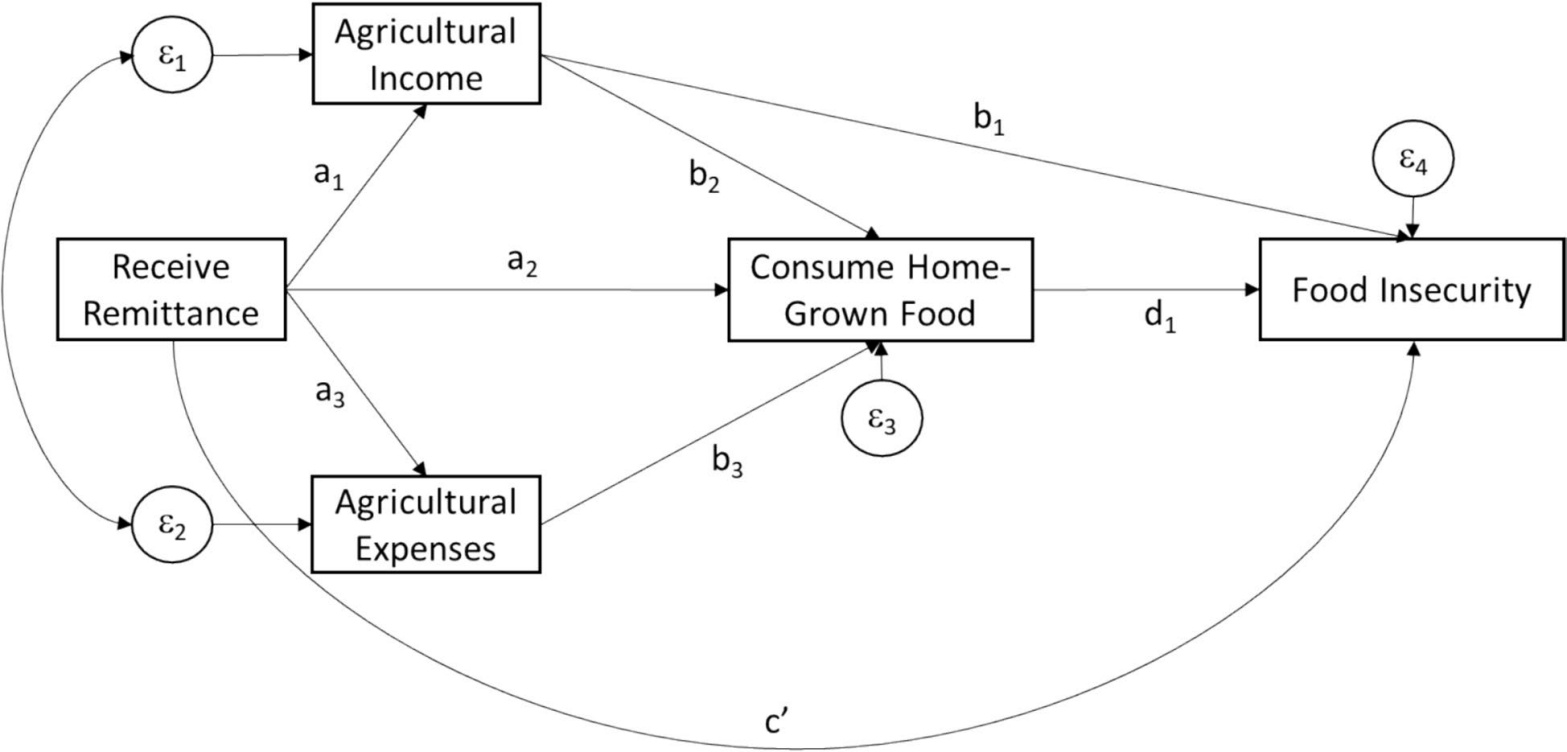
Path diagram of agricultural mechanisms of remittances and food insecurity. Remittances predict agricultural income (path a_1_), the likelihood to consume home-grown foods (path a_2_), and agricultural investments (path a_3_). Income and expenses related to agriculture predict the likelihood of consuming home-grown foods (paths b_2_ and b_3_). Since income and expenses in the previous year are bidirectional the residuals of the two variables covary. Food security is predicted by accessibility enabled by consumption of home-grown foods (path d_1_), and food purchased through the market with agricultural income (path b_1_) and remittance income (path c’)

The path diagram is depicted in Fig. 1. Among households engaged in crop and/or livestock cultivation, remittances predict agricultural income (path a_1_), the likelihood to consume home-grown foods (path a_2_), and agricultural investments (path a_3_). Income and expenses related to agriculture predict the likelihood of consuming home-grown foods (paths b_2_ and b_3_). Since income and expenses in the previous year are bidirectional (that is, they both can predict each other), we specify that the residuals of the two variables covary. Food security is predicted by accessibility enabled by consumption of home-grown foods (path d_1_), and food purchased through the market with agricultural income (path b_1_) and remittance income (path c’).

The main effects of interest are: (1) the total effect of remittances on the likelihood of consuming home-grown foods via the direct pathway and indirect pathways through agricultural income and expenses (a_1_*b_2_ + a_3_*b_3_ + a_2_); (2) the indirect effect of remittances on food insecurity via the agricultural income and production pathway (all paths except c’); and (3) the total effect of remittances on food insecurity via the agricultural and market pathways (all paths).

All regression equations implied in the model control for the same variables listed in Sect. "Data source and variables", except instead of a binary measure of animal ownership, we include number of animals reported to be owned by the household. The serial mediation analysis was run in R using the ‘lavaan’ package with the diagonally weighted least squares method to estimate standardized and unstandardized coefficients [27]. Model fit was assessed using goodness-of-fit measures, including the model chi-square test, the comparative fit index (CFI), the Tucker–Lewis index (TLI), the root mean square error of approximation (RMSEA), and the standardized root mean squared residual (SRMR) [28].

All analyses account for the complex sampling design of IHDS.

## Results

### Remittances and food insecurity

12.8% of households receive remittances from a migrant member. This varies by demographic characteristics (Table 1). A disproportionately higher percentage of rural, smallholder, and livestock-rearing households receive remittances. The overall prevalence of food insecurity is close to 15%, with lower income and lower education, rural, smallholder, marginalized and minority social groups, and woman-headed households experiencing higher than average food insecurity.

**Table 1:** Sociodemographic characteristics, remittance status, and food insecurity prevalence, 2011–12, *n* = 39,292

	All^1^		Receives remittances^1^			Food insecure^2^		
	%	SE	%	SE	*p*	%	SE	*p*
Social group								
Forward caste	19.67	0.62	19.73	1.13		10.37	0.76	
OBC	35.92	0.86	40.68	1.87		11.95	0.60	
Dalit	23.08	0.70	20.83	1.36		18.34	0.98	
Adivasi	8.16	0.55	5.85	0.64		22.46	2.05	
Muslim	11.08	0.64	11.78	1.13		21.61	1.43	
Other	2.06	0.23	1.13	0.21	< 0.001	3.48	0.80	< 0.001
Income quintile								
Lower	19.75	0.41	21.13	0.96		21.55	1.00	
Second	19.74	0.32	20.58	0.95		20.39	1.04	
Middle	19.82	0.28	21.28	0.80		15.93	0.71	
Fourth	19.75	0.32	21.23	1.02		11.02	0.59	
Higher	19.67	0.43	15.78	0.75	< 0.001	5.71	0.40	< 0.001
Location								
Rural	73.86	0.68	86.44	0.82		17.90	0.67	
Urban	26.14	0.68	13.56	0.82	< 0.001	6.29	0.41	< 0.001
Land size								
None	51.40	0.72	39.96	1.60		14.29	0.58	
< 2 hectares	41.31	0.71	54.01	1.71		16.58	0.70	
≥ 2 hectares	7.28	0.30	6.03	0.57	< 0.001	9.21	0.85	< 0.001
Livestock ownership								
No	54.42	0.73	41.67	1.50		12.33	0.52	
Yes	45.58	0.73	58.33	1.50	< 0.001	17.90	0.67	< 0.001
Household size								
1–3	27.54	0.46	38.34	1.23		12.45	1.28	
4–6	53.91	0.37	42.87	1.10		13.87	0.56	
7–9	14.55	0.36	13.22	0.84		15.51	0.87	
≥ 10	4.01	0.17	5.57	0.54	< 0.001	12.45	1.28	< 0.001
Gender of head of household								
Man	83.84	0.35	63.17	1.31		14.24	0.52	
Woman	13.97	0.32	36.83	1.31	< 0.001	18.71	0.97	< 0.001
Highest education level								
None	20.30	0.48	32.95	1.19		25.12	1.09	
1–5 y	14.43	0.34	16.43	0.87		20.82	1.03	
6–10	35.49	0.48	29.07	1.23		13.89	0.58	
11–14	14.55	0.33	11.17	0.66		8.23	0.56	
Bachelor or higher	15.20	0.40	10.38	0.63	< 0.001	4.17	0.42	< 0.001
Ration card usage								
No	21.32	0.58	15.34	0.82		14.75	0.74	
Yes	78.62	0.58	84.66	0.82	< 0.001	14.90	0.56	0.851
Total	100.00		100.00			14.87	0.51	

^1^ column percentage, may not add up to 100 due to missing values

^2^ row percentage

*SE* standard error

In 2011–12, per capita food consumption among households that began receiving remittances is on average Rs. 3277 (95% CI 3174–3380), compared to Rs. 3607 (95% CI 3547–3667) among households that never received remittances. In the adjusted model, however, at the same income level, and controlling for prior food consumption, remittance receiving households consume three percent more food (Table 2a), and are 18% less likely to be food insecure compared to non-remittance receiving households (Table 2b). Other predictors of higher food consumption and lower food insecurity are per capita income and education.

**Table 2:** Adjusted estimates of food consumption and food insecurity, 2004–05, 2011–12

	(a) Per capita food consumption (log)		(b) Food insecurity	
	exp(β)	95% CI	OR	95% CI
Receives remittances	1.03	(1.01–1.05)	0.82	(0.71–0.95)
Per capita income (log)	1.12	(1.10–1.14)	0.89	(0.85–0.92)
Social group				
Forward caste	Ref	Ref	Ref	Ref
Other backward class	0.95	(0.92–0.97)	0.97	(0.81–1.15)
Dalit	0.91	(0.88–0.93)	1.26	(1.06–1.49)
Adivasi	0.85	(0.82–0.89)	1.40	(1.08–1.82)
Muslim	1.03	(0.99–1.06)	1.41	(1.15–1.72)
Other	1.00	(0.95–1.05)	0.61	(0.34–1.07)
Urban location	1.15	(1.12–1.18)	0.46	(0.39–0.54)
Farm size				
None	Ref	Ref	Ref	Ref
< 2 hectares	1.04	(1.03–1.06)	0.92	(0.82–1.04)
≥ 2 hectares	1.13	(1.10–1.17)	0.76	(0.60–0.95)
Animal ownership	1.08	(1.06–1.10)	1.28	(1.14–1.42)
Ration card usage	0.94	(0.92–0.96)	0.75	(0.66–0.85)
Woman head of household	0.94	(0.93–0.96)	1.30	(1.12–1.50)
Highest education level				
None	Ref	Ref	Ref	Ref
1–5 years	1.05	(1.02–1.07)	0.83	(0.73–0.95)
6–10 years	1.13	(1.10–1.15)	0.63	(0.56–0.72)
11–14 years	1.17	(1.15–1.20)	0.43	(0.37–0.52)
Bachelor or higher	1.30	(1.26–1.34)	0.28	(0.22–0.36)
Dependency ratio	0.96	(0.95–0.97)	1.08	(1.02–1.14)
2006 food consumption	1.16	(1.13–1.18)		
2006 food share			1.01	(1.01–1.01)
Observations	37,983		37,783	
R-squared	0.38			

Models additionally control for state of residence. *OR* Odds ratio, *CI* Confidence interval

Smallholders have the same food consumption and odds of food insecurity compared to landless households, while medium or larger landholdings are associated with higher food consumption and lower food insecurity.

### Mechanisms of remittances and food insecurity

#### Crop production

**Fig. 2:**
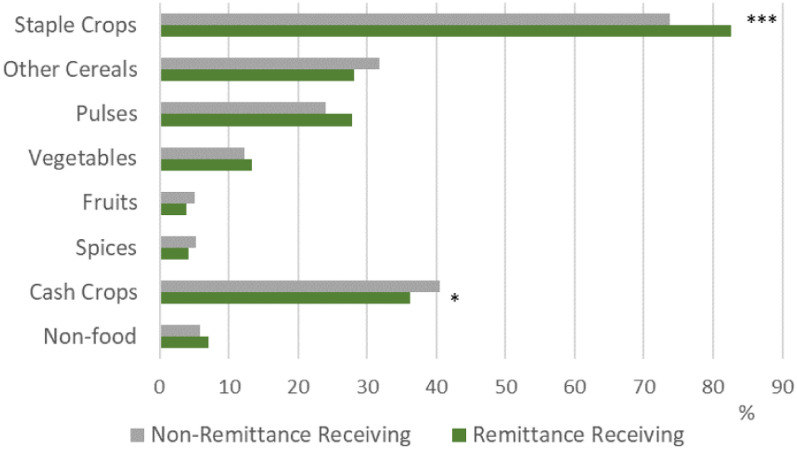
Crop production among farm-cultivating households, 2011–12 *n* = 14,903 ***p < 0.001; **p < 0.01; *p < 0.05

As shown in Fig. 2, staple crop production, including rice and wheat, was higher among remittance receiving households (83%), compared to non-remittance receiving households (74%). Conversely, production of commercial cash crops, including cotton, sugarcane, tobacco, coffee, tea, oilseeds, etc., was higher among non-remittance receiving households. All other categories of crops were similar between the two groups.

#### Consumption of foods from home production

71% of agricultural remittance receiving households consumed food produced at home in the previous month, compared to 65% of non-remittance receiving households (Table 3). Among land-cultivating households, those that receive remittances are more likely to consume home-grown staples during the previous month, including rice, wheat, and other cereals, while there is no difference between the two groups for pulses and vegetables. Among livestock-rearing households, there is no difference in home-produced food consumption between remittance and non-remittance receiving households for most animal source foods, except eggs, which have a slightly higher consumption among non-remittance receiving households.

**Table 3:** Consumption of home-grown foods in the previous 30 days by remittance status, 2011–12

	Non-remittance households		Remittance households		*p*
	%	SE	%	SE	
Any crop^1^	63.43	1.05	71.91	1.40	< 0.001
Staple	54.42	1.16	62.55	1.72	< 0.001
Other cereal	35.79	1.75	50.49	3.27	< 0.001
Pulses	9.94	0.81	10.17	1.32	0.863
Vegetables	6.15	0.52	5.05	0.69	0.156
Any ASF^2^	47.20	1.07	47.28	1.69	0.961
Meat	2.22	0.24	1.60	0.40	0.183
Egg	8.03	0.82	5.55	1.01	0.049
Milk product	54.96	1.05	55.24	2.06	0.883
Any food^3^	65.49	0.91	71.22	1.28	< 0.001

^1^among land-cultivating households, *n* = 18,283

^2^among livestock-owning households, *n* = 17,168

^3^among land-cultivating or livestock-owning households, *n* = 22,297

*ASF* animal source foods, *SE* standard error

**Fig. 3:**
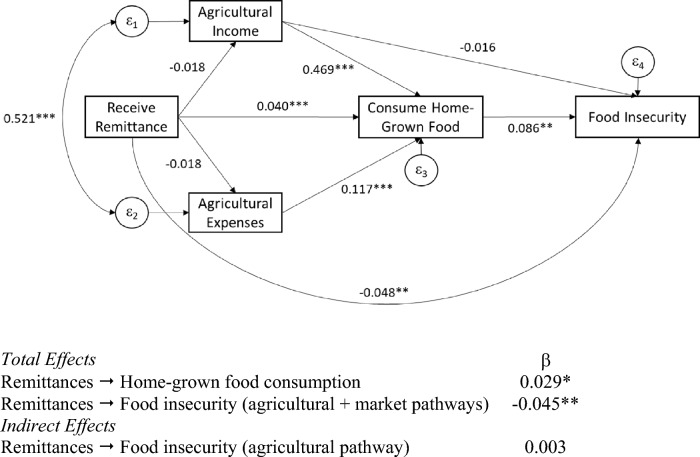
Standardized coefficients of the pathways from remittances to food security through agriculture and market mechanisms. Goodness-of-fit measures: *χ*^2^:0.043, df = 1, *p* = 0.836; CFI: 1.00; TLI: 1.00; RMSEA: 0.00; SRMR: 0.00

Correlations of the main serial mediation model variables are reported in Additional Table 1. Our hypothesized model fits the data well; goodness-of-fit statistics are reported in Fig. 3, as are standardized coefficients of direct effects, and indirect and total effects of interest. Unstandardized coefficients are reported in Additional Table 2.

Agricultural households that began receiving remittances before or during the 2011–12 wave spent the same on agriculture-related expenses, and earned the same gross agricultural income as households that never received remittances, controlling for land size and other covariates. Consumption of home-grown foods in the previous month was positively predicted by receiving remittances (*β* = 0.040; *p* < 0.001), agricultural income (*β* = 0.469; *p* < 0.001), and agricultural expenses (*β* = 0.117; *p* < 0.001) in the previous year.

Receiving remittance income in the previous year predicted lower food insecurity in the previous month (*β* = −0.048; *p* = 0.001). Controlling for remittance status, higher agricultural income had no effect on food insecurity, while consumption of home-grown food predicted higher food insecurity (*β* = 0.086; *p* = 0.001).

The total effect of receiving remittances on the likelihood of consuming food from home production remains positive and significant (*β* = 0.029; *p* = 0.020) despite the negative indirect effect that approaches significance through agricultural income (*β* = −0.008; *p* = 0.066). Receiving remittances had a negative total effect on the likelihood of food insecurity (*β* = −0.045; *p* = 0.002); the total effect was not significantly mediated by the agricultural pathways of production, income, and consumption of home-grown food (*β* = 0.003; *p* = 0.063), but rather, explained by the direct effect of remittances on food insecurity, that is, the market pathway.

## Discussion

This study adds to the existing evidence on the migration–food security nexus. We find that households at the same total income level that begin to receive remittances from internal migrant members consume more food per capita compared to households that do not receive remittances. Furthermore, the share of total consumption that is allocated to food is lower among remittance receiving households; these findings together suggest overall improved food security due to remittances.

This study sheds light on the thus far hypothesized mechanisms of remittances to food security. Among agricultural households, that is households that cultivate land and/or rear livestock, we found that the agricultural pathway does not explain improved food security observed for remittance receiving households. In the first stage of the pathway, we find that remittance receiving agricultural households do not earn more or less income from agriculture compared to non-remittance receiving households. While the widely held policy perspective is that migration negatively impacts agricultural productivity and income due to labor losses in rural origin areas, our findings align with de Brauw’s conclusion to the contrary from his review on the impacts of migration on rural livelihoods [29]. Rather, sending households are able to either adjust production techniques or production patterns to maintain similar agricultural income levels. If the former were the case, we would expect to observe higher expenses among remittance receiving households for agricultural investments, such as hiring of labor to replace migrant members, or adoption of labor-saving technologies. However, we find that remittance receiving households are no more or less likely to invest in agriculture compared to non-remittance receiving households, when taken together with our findings on income, imply adaptations that do not require investments, for example, renting out land, or replacing labor-intensive crops with less labor-intensive crops [30].

While agricultural income and expenses remain similar, consumption of home-produced food is higher among households that began receiving remittances in 2011–12 compared to households that never received remittances. However, this pathway does not contribute to improved food security. Rather, it is the market source that explains improved food security among remittance receiving households. This is perhaps not surprising given we observed that remittance receiving households are more likely to produce and consume staple foods of rice and wheat, which must be supplemented by market purchases. This has important food systems policy implications. The program prescriptive to address food insecurity among smallholder farmers is often through interventions, such as production diversity, which could include, for example, integrating additional crops into existing farming systems, crop rotations, or combining crops and livestock. Under the umbrella scheme for the development of agricultural and allied sectors, Rashtriya Krishi Vikas Yojana, the Government of India implements a crop diversification program in the Green Revolution states of Haryana, Punjab, and Uttar Pradesh, as well as several tobacco producing states. In addition to the environmental sustainability benefits of crop diversification, such as soil health and protection from pests, evidence supports a negative effect of crop diversification on poverty among Indian farmers [31].

A critical factor in whether product diversification can alleviate poverty is access to markets [32]. For rural households with migrant members, this may create further barriers depending upon the extent of market integration of family members who remain in source areas. In addition to labor, those members of the household who migrate are potentially the primary decision-makers related to agricultural production [29] and may be the family’s representative in the market. Our results show that while around 14% of households overall in India are woman-headed, this figure is over 2.5-fold (37%) among remittance receiving households. Heavier workloads faced by women in agricultural out-migrant households [33], coupled with gender norms in the South Asian context surrounding women’s mobility and domestic responsibilities, can restrict women’s engagement in the market. Indeed, our results demonstrate that woman-headed households experience higher food insecurity and consume less food holding remittance status constant, potentially implying restraints to market access for both purchasing and selling foods. Interventions such as community marketing to link women to markets may be promising [34].

Seasonality presents another reason a crop diversification strategy must be sensitive to migration patterns. There is a large literature on crop diversification and its effects on dietary diversity [35], an indicator of food security that addresses diet quality. Studies have overall found a positive, albeit marginal, association between production diversity and dietary diversity. However, a more nuanced examination considering seasonality finds that planting multiple crops within a year results in higher dietary diversity during the monsoon season [36], recommending intensification of diversification strategies during the winter and summer agricultural seasons. There is a seasonality to many streams of migration as well; for example, the construction industry, the leading employer of short-term migrants from rural areas [37], is at peak operation during these same seasons. Therefore, the policy call based on the conclusion that making healthy foods more affordable and accessible through strong rural markets is more effective for improving diets and food security than crop diversification [35, 38] is even more salient for households with migrant members.

A major limitation to this study is the measure of food insecurity used based on the available data in IHDS; household food consumption share does not fully address the multiple dimensions of food insecurity, such as preferred foods, or dietary quality. Therefore, although we found that remittance receiving households consume more food overall and are more likely to consume home-produced food, since the foods produced and consumed at home are primarily staples, a critical question becomes what foods are being purchased in the market? Research from the region suggests that remittances are used to purchase both healthy foods such as meat and fish [39] as well as unhealthy foods such as sugary beverages [40]. An ideal assessment of food security would include an experience-based scale such as the Food Insecurity Experiences Scale [41] coupled with household dietary diversity and individual dietary diversity measured using a food frequency questionnaire or a dietary quality screening tool to also understand intra-household allocation of food. Especially among communities who are dependent on movement and land for livelihood, there is a need for measurement of multiple dimensions of food insecurity, including consumptive, experiential, and economic measures, to capture nuanced coping strategies in the face of environmental and economic shocks [42]. In addition, IHDS largely relies on self-reported data; variables capturing monetary amounts over a 1-year period such as agricultural expenditures and agricultural income are subject to recall bias.

This study had several strengths. We utilized longitudinal data to understand how households that began to receive remittances after 2004–05 but before 2011–12 differ from households that never received remittances. In addition, we focused exclusively on remittances from internal migration, which in the context of India is arguably more significant than international migration as a result of the magnitude of internal movement and households affected by migration. Indeed, unlike international migration, internal labor migration in India is characterized by unaccompanied men out-migrating, thereby affecting household composition, livelihoods, and gender norms and relations in the origin. We would expect similar, if not stronger, results for the effect of international remittances on food security. In a study of 92 (LMIC), international, domestic, and remittances from a combination of both were all positively associated with improved food security; however, international remittances had the largest effect [43]. We might expect differing results with respect to the effect of international remittances on agricultural investment; studies from Bangladesh [44] and China [45], for example, have found no effect of domestic remittances on agricultural investment, similar to our findings. However, the same study from Bangladesh, as well as evidence from Mexico [46] has found a positive effect of international remittances on agricultural investment. Further research is required in India.

### Policy implications

Our findings have important policy implications for rural food systems in two key areas. First, among agricultural households, rural markets are a significant source for non-staple nutritious foods, such as vegetables, fruits, pulses, and animal source foods. Thus, making healthy foods affordable is crucial, perhaps more so than promoting crop diversification for food security. Strategies to improve affordability of healthy diets can include shifting producer and consumer subsidies from staple crops, such as rice and wheat, to more nutrient-dense foods, such as fruits and vegetables. Investment into required infrastructure to minimize post-harvest loss is another policy imperative to reduce costs of healthy and perishable foods. This includes, for example, building of roads for efficient transport, and setup of cold storage and warehousing facilities [47].

Secondly, for agricultural households with members who migrate for part or all of the year, efforts to link remaining members to markets must be strengthened, especially for households in which women become the de facto head due to the migration of men, who, even in the case of improved infrastructure mentioned above, may face bias in market access [48]. Aggregation initiatives can address the economies of scale problem that many smallholders face in linking to the market [49]. Examples of successful collective farming models for women farmers, such as land pooling, joint investment, and collective cultivation, would require policy intervention in the form of, for example, subsidized credit, or group land rights [48]. These models are also relevant for other LMIC contexts with high male outmigration.

### Conclusion

In conclusion, this study adds to the existing evidence that remittances improve food security, and is the first to contribute a test of the hypothesized agricultural mechanisms. We find that for agricultural households, the primary mechanism for remittances to improve food security is the market pathway. For agricultural households that receive remittances, interventions to improve production need to be migration sensitive, and policies must prioritize strengthening rural markets to make healthy food affordable for all.

## Supplementary Information

Supplementary material 1.

## Data Availability

India Human Development Survey data are publicly available at the Inter-university Consortium for Political and Social Research (ICPSR) repository.

## Footnotes

1 Defined as a household receiving value of produce more than Rs. 4000/- from agricultural activities and having at least one member self-employed in agriculture either in the principal status or in subsidiary status during last 365 days.

2 Home-grown food could also include gifted food from another household.

3 Dalits are Scheduled Castes and refer to those who have been marginalized due to the Hindu caste hierarchy; Adivasis are Scheduled Tribes and refer to those who have been marginalized due to geographical isolation. Other Backward Classes (OBC) are communities that are economically, socially or educationally disadvantaged and do not classify as Dalit or Adivasi.
