# Supplementary material for: The pathway from domestic remittances to food security in India: is agriculture a mediator?
Source: Agric Food Secur. 2026 Mar 22;14(1):49. doi: 10.1186/s40066-025-00574-9 (PMC13005802; doi:10.1186/s40066-025-00574-9)
Supplement: Supplementary file 1 — Supplementary material 1. [file 40066_2025_574_MOESM1_ESM.docx]

**Additional Table 1: Polychoric, polyserial and Pearson correlation matrix of study variables**

|  | 1 | 2 | 3 | 4 | 5 |
| --- | --- | --- | --- | --- | --- |
| 1. Receives Remittances | 1.000 |  |  |  |  |
| 2. Agriculture Expense (log) | -0.043 | 1.000 |  |  |  |
| 3. Agriculture Income (log) | -0.028 | 0.575 | 1.000 |  |  |
| 4. Consume Home-Grown Food | 0.084 | 0.487 | 0.637 | 1.000 |  |
| 5. Food Insecurity | 0.003 | -0.054 | -0.054 | 0.055 | 1.000 |

*Column heading codes correspond to row heading code definitions*

**Additional Table 2: Standardized and unstandardized coefficients of pathways from remittances to food security through agriculture and market mechanisms: direct and total effects**

|  | Dependent Variables | | | | | | | | | | | |
| --- | --- | --- | --- | --- | --- | --- | --- | --- | --- | --- | --- | --- |
|  | Agricultural Expense (log) | | | Agricultural Income (log) | | | Consumption of Home-Grown Foods | | | Food Insecurity | | |
|  | *b* | *SE* | *β* | *b* | *SE* | *β* | *b* | *SE* | *β* | *b* | *SE* | *β* |
| **Direct Effects** |  |  |  |  |  |  |  |  |  |  |  |  |
| Receives Remittance | -0.159 | 0.108 | -0.018 | -0.170 | 0.092 | -0.018 | 0.135*** | 0.037 | 0.040 | -0.157** | 0.047 | -0.048 |
| Agricultural Expense |  |  |  |  |  |  | 0.045*** | 0.005 | 0.117 |  |  |  |
| Agricultural Income |  |  |  |  |  |  | 0.166*** | 0.003 | 0.469 | -0.005 | 0.007 | -0.016 |
| Consumption of Home-Grown Foods |  |  |  |  |  |  |  |  |  | 0.083** | 0.027 | 0.086 |
| **Total Effects** |  |  |  |  |  |  |  |  |  |  |  |  |
| Agriculture Pathway |  |  |  |  |  |  | 0.099* | 0.043 | 0.029 | 0.009 | 0.005 | 0.003 |
| Agriculture + Market Pathway |  |  |  |  |  |  |  |  |  | -0.147** | 0.047 | -0.045 |

*Restricted to HH that cultivate land and/or own livestock (n=21,142)*

** <0.05; ** <0.01; *** <0.001*

*SE: standard error; β: standardized coefficient; b: unstandardized coefficient*
